# Cross-talks via mTORC2 can explain enhanced activation in response to insulin in diabetic patients

**DOI:** 10.1042/BSR20160514

**Published:** 2017-01-25

**Authors:** Rasmus Magnusson, Mika Gustafsson, Gunnar Cedersund, Peter Strålfors, Elin Nyman

**Affiliations:** 1 Department of Biomedical Engineering, Linköping University, Sweden; 2 Department of Physics, Chemistry and Biology, Linköping University, Sweden; 3 Department of Clinical and Experimental Medicine, Linköping University, Sweden; 4 CVMD iMed DMPK AstraZeneca R&D, Gothenburg, Sweden

**Keywords:** Type 2 diabetes, mTORC1, protein kinase B, computational models, SIN1, mTORC2

## Abstract

The molecular mechanisms of insulin resistance in Type 2 diabetes have been extensively studied in primary human adipocytes, and mathematical modelling has clarified the central role of attenuation of mammalian target of rapamycin (mTOR) complex 1 (mTORC1) activity in the diabetic state. Attenuation of mTORC1 in diabetes quells insulin-signalling network-wide, except for the mTOR in complex 2 (mTORC2)-catalysed phosphorylation of protein kinase B (PKB) at Ser^473^ (PKB-S473P), which is increased. This unique increase could potentially be explained by feedback and interbranch cross-talk signals. To examine if such mechanisms operate in adipocytes, we herein analysed data from an unbiased phosphoproteomic screen in 3T3-L1 adipocytes. Using a mathematical modelling approach, we showed that a negative signal from mTORC1-p70 S6 kinase (S6K) to rictor–mTORC2 in combination with a positive signal from PKB to SIN1–mTORC2 are compatible with the experimental data. This combined cross-branch signalling predicted an increased PKB-S473P in response to attenuation of mTORC1 – a distinguishing feature of the insulin resistant state in human adipocytes. This aspect of insulin signalling was then verified for our comprehensive model of insulin signalling in human adipocytes. Introduction of the cross-branch signals was compatible with all data for insulin signalling in human adipocytes, and the resulting model can explain all data network-wide, including the increased PKB-S473P in the diabetic state. Our approach was to first identify potential mechanisms in data from a phosphoproteomic screen in a cell line, and then verify such mechanisms in primary human cells, which demonstrates how an unbiased approach can support a direct knowledge-based study.

## Introduction

Type 2 diabetes is a metabolic disease that is closely related to overweight and obesity. At the heart of the disease is a dysfunction in the intracellular mechanism of insulin signalling in adipose, muscle and liver tissues. This dysfunction is referred to as insulin resistance but the underlying molecular mechanisms are not fully understood. A reason for this lack of understanding is the complexity of the intracellular transmission of insulin signals via multiple protein interactions. While the core of the insulin-signalling network is well established, details regarding feedbacks and cross-talks are yet to be discovered. To relate the importance of such discoveries to insulin resistance in Type 2 diabetes, a systematic analysis of data from relevant cells are required.

We have previously collected dynamic, systems-wide, internally consistent data on insulin-signalling proteins from the same cells: primary human adipocytes obtained from both normal non-diabetic subjects and obese patients with Type 2 diabetes [1,2]. These experimental studies were combined with mathematical modelling in a systems approach to compare the insulin-signalling network in the normal state with the same network in the insulin-resistant state of diabetes [1,2]. The main result was that attenuation of insulin signalling in Type 2 diabetes is explained by an attenuated feedback from the mammalian target of rapamycin (mTOR) complex 1 (mTORC1) to phosphorylation of insulin receptor (IR) substrate-1 (IRS1) at Ser^307^ (human sequence) [1–4] in combination with reduced abundance of IR, glucose transporters (GLUT4), the ribosomal protein S6, AS160 and FOXO1 [5]. This model [1,2,5] is the first model of insulin signalling that is based on molecular data from patients with insulin resistance and Type 2 diabetes, which is designed to understand the disease. For the insulin-signalling network normally, several models have been developed over the years (reviewed in [6,7]).

**Figure 1:**
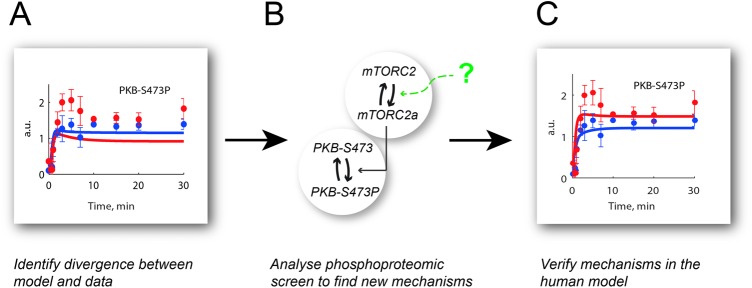
Modelling workflow (**A**) This project was initiated by a lack of agreement between simulations of a previously developed model for insulin signalling and corresponding experimental data for PKB-S473P in primary human adipocytes obtained from diabetic patients. (**B**) We used data from a phosphoproteomic screen in response to insulin in 3T3-L1 adipocytes to test new mechanisms that could potentially explain the human PKB-S473P data. (**C**) We verified the mechanisms from (B) in the model for human adipocytes in diabetes with a resulting acceptable fit with data for PKB-S473P.

Our previously developed model for the diabetic state [1,2] was generally congruent with experimental data, with a single exception: phosphorylation of protein kinase B (PKB) at Ser^473^ (PKB-S473P) (Figure 1A). This residue in PKB is phosphorylated by mTOR in complex with rictor (mTORC2) [8,9]. In contrast with a marked decrease in the phosphorylation of PKB at Thr^308^, both in data and model simulations, our data indicated an increase in PKB-S473P in adipocytes from diabetic patients (Figure 1A, red dots), whereas model simulations showed a small decrease in PKB-S473P (Figure 1A, red line). This unusual regulation of PKB-S473 is more poignant as inhibition of mTORC1 with rapamycin induces a reduced response to insulin in almost all observed signalling intermediaries in human adipocytes [1,10]; one of the few exceptions is the PKB-S473P, where rapamycin instead induces a small increase [1]. This discrepancy between experimental data and model simulations indicates that mechanisms are lacking in the model structure, upstream or at the level of PKB-S473P.

Potential mechanisms that can affect PKB-S473P via its kinase mTORC2 have been described in various cell types. The mTORC2 complex consists of several proteins, of which some are unique for mTORC2, e.g. SIN1 and rictor. SIN1 can be phosphorylated at Thr^86^ and Thr^398^, and PKB [11,12] and p70 S6 kinase (S6K) [12,13] have been implicated in this process. Moreover, mTORC2 can also be inhibited via phosphorylation of rictor at Thr^1135^, supposedly by S6K [14–16]. Phosphorylation of SIN1 has potentially both positive and negative effects on the kinase activity of mTORC2 [13,17], whereas phosphorylation of rictor has been found to be a negative regulator [14]. These mechanisms can theoretically result in different effects on the kinase activity of mTORC2 and therefore also for the resulting PKB-S473P. However, these mechanisms have not been quantifiably evaluated to examine how they together fit into the dynamics of insulin signalling in adipocytes.

Herein, we have analysed possible underlying mechanisms for the increased PKB-S473P that we find in adipocytes from patients with Type 2 diabetes [1]. The data from our earlier studies of human adipocytes do not include dynamic measurements of the proteins of the mTOR 1 and 2 complexes, and therefore cannot be used to test hypotheses regarding detailed mechanisms that involve these complexes. Such data are, to the best of our knowledge, not available, and we instead extracted data from a phoshoproteomics screen of the insulin response in 3T3-L1 adipocytes [11]. We used these data with mathematical modelling to evaluate possible mechanisms and found that a combined positive and negative input to mTORC2 can produce the increase in PKB-S473P upon inhibition of mTORC1, which mimics the diabetic state. We introduced this mechanism in our comprehensive model of the insulin-signalling network in human adipocytes (Figure 1B). The resulting model provides a better fit with PKB-S473P data in the diabetic state than the original model (Figure 1A) – while still fitting the rest of the experimental data from the human adipocytes.

## Results

### Analysis of the diabetic state of our comprehensive model of insulin signalling in human adipocytes

**Figure 2:**
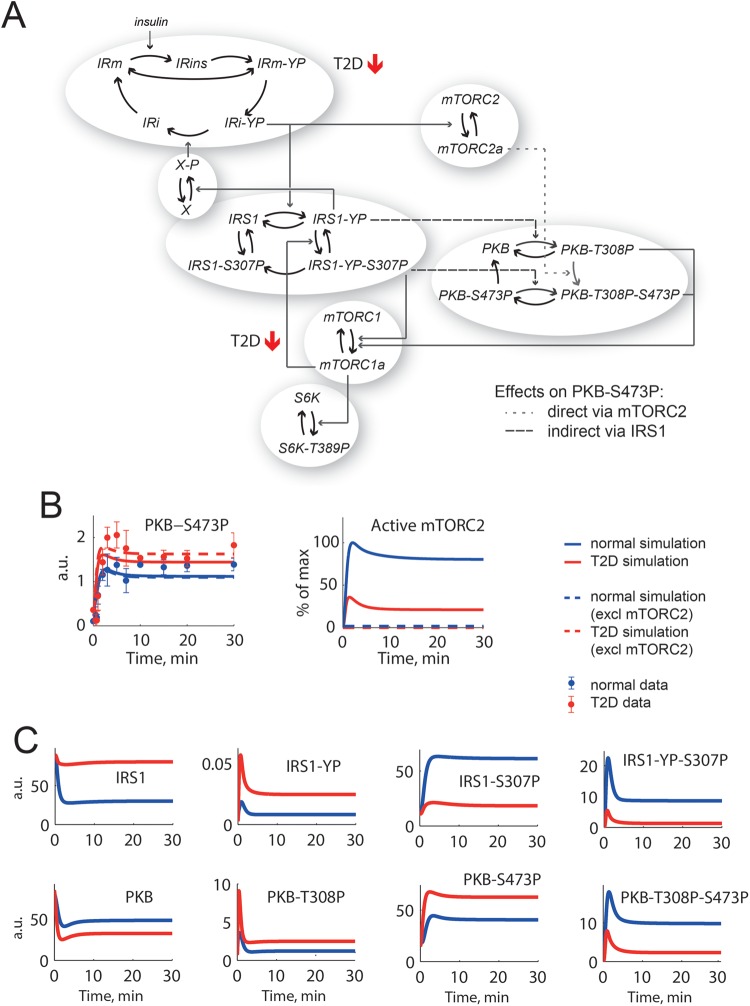
A core model of insulin signalling in human adipocytes can be forced to simulate PKB-S473P in the diabetic state, but with unacceptable consequences (**A**) The diabetes model from [1,2] reduced to a core network with IR, IRS1, PKB, mTORC1 and mTORC2, p70 S6K and a negative feedback to IR via an unknown protein X [19 ]. The insulin resistance is induced in this model by changing two parameters: total amount of IR is down to 55% and the feedback from mTORC1 to IRS1 is attenuated (marked with red arrows). Marked with a dotted line is the direct effect from mTORC2 to PKB-S473P and with dashed lines are the indirect effects from IRS1 that affects PKB-S473P. (**B**) To the left, data from primary human adipocytes for PKB-S473P in response to insulin normally (blue dots ± S.E.M.) and in diabetes (red dots ± S.E.M.) compared with corresponding model simulations (lines). To the right, the corresponding simulations for mTORC2 (lines). Simulations are chosen for best possible fit with PKB-S473P and acceptable fit with other data. The direct effect from mTORC2 to PKB-S473P is nearly absent, which becomes obvious when mTORC2 is completely inhibited in the model (dashed lines). (**C**) Simulations of the individual states of IRS1 and PKB reveal that single phosphorylated IRS1-YP and PKB-T308P have a higher response in diabetes (red) than normal (blue), but total IRS1-YP and PKB-T308P have a lower response in diabetes since the double phosphorylated states (IRS1-YP-S307P and PKB-T308P-S473P) are dominating.

We first analysed the original model of the insulin-signalling network in human adipocytes [1,2] with emphasis on the ability of the model to produce an increased response at PKB-S473P in the diabetic state (Figure 1A). We used a stripped-down model structure with only the core proteins of the signalling network, i.e. IR, IRS1, PKB, S6K, mTORC1 and mTORC2 (Figure 2A). Model parameters were reoptimized with an extra penalty added to deviation from this specific data set (PKB-S473 in diabetic cells; see ‘Materials and methods’).

This analysis revealed that it was possible to find parameters that can produce the increased response at PKB-S473P with the original model structure (Figure 2B, red solid line). However, further analysis revealed that the increase was due to the model structure with IRS1 and PKB exhibiting four interconnected states each, which correspond to non-phosphorylated, single-phosphorylated and double-phosphorylated IRS1/PKB respectively (Figure 2A). Simulations of all these states (Figure 2C) also show that single-phosphorylated IRS1-YP and PKB-T308P are increased in the diabetic state even though the dominating double-phosphorylated (IRS1-YP-S307P and PKB-T308P-S473P) are not. For these parameters, the dynamic interplay between PKB and IRS1 states gives a solution where IRS1 inputs indirectly give rise to the increase in PKB-S473P (Figure 2A, dashed arrow). At the same time, there is hardly any direct effect of mTORC2 on PKB-S473P with this parameter solution (Figure 2A, dotted arrow) and mTORC2 can even be excluded from the model (Figure 2B, dashed lines). However, mTORC2 is known to be the protein kinase responsible for PKB-S473P in response to insulin [8,9], and the simulations of PKB-S473P should therefore be affected when mTORC2 is removed from the system. We thus deemed that a negative signal to mTORC2 is missing in the original model structure.

To improve the model of insulin signalling to better fit with the observed increase in PKB-S473P in the diabetic state, we sought realistic modifications to the model structure in a two-step approach: (i) using phosphoproteomics data obtained from 3T3-L1 adipocytes [11] to examine possible signals (Figure 1B and Table 1) and (ii) using this knowledge to develop a new version of the model of insulin signalling, which can also explain the increased PKB-S473P in the diabetic state of human adipocytes (Figure 1C).

**Table 1:** Summary of tested hypotheses for control of mTORC2

	Experimental observations			
	3T3-L1 adipocytes [11]		3T3-L1 and human adipocytes [39,40]	Human adipocytes [1]
Tested mechanism	Time-series data	PKB-inhibition data	PKB-S473P increase with mTORC1 inhibition	Time-series data
PKB → SIN1 (positive)	Ok	Ok	Fail	
PKB → SIN1 (negative)	Ok	Ok	Fail	
S6K → rictor (negative)	Ok	Ok	Ok	Fail
PKB → SIN1 (positive) +	Ok	Ok	Ok	Ok
S6K → rictor (negative)				

### Identification of mechanisms of mTORC-signalling based on data from 3T3-L1 adipocytes

We extracted data of protein phosphorylation involved in mTORC-signalling in response to insulin in 3T3-L1 adipocytes from the published phosphoproteomic database [11]. For some of the phosphorylation sites, data were available (e.g. SIN1-T86P and PKB-S473P) in the database, but not for all the sites. We therefore used available data for less studied phosphorylation sites (e.g. S6K-S427P/452P) as proxies for the corresponding protein activities. In those cases, we chose phosphorylation sites that responded both to insulin and to inhibitors (MK2206 and LY294002) used in the study [11] (see ‘Materials and methods’).

First, we tested if the extracted data set from 3T3-L1 adipocytes was an acceptable proxy for the human insulin-signalling system, using correlation analysis. Four of the extracted data sets (IR-YP, IRS1-YP, PKB-S473 and S6K-P) could be directly compared with corresponding human data from [1]. Out of these four, three displayed a significant positive Spearman correlation coefficient (*P*<0.05). The fourth, IRS1-YP, displayed a marginal positive correlation (*P*=0.12). The shapes of the time-course responses were similar with a distinct peak followed by an enhanced steady-state level for both the 3T3-L1 and human cells, but the peak comes earlier in the murine 3T3-L1 than in the human data. We have earlier identified a faster peak response of IRS1-YP in rat compared with human adipocytes [18]. As this difference is not the focus of our present investigation, we excluded the IRS1-YP data from the present study. Furthermore, the three additional proteins present in both studies were found to have a significant positive correlation between the two studies (ERK1–T202P–Y204P, AS160–T642P and S6–S235/236P). To summarize, the protein responses to insulin in the two studies showed quantifiably the same dynamics (binomial test *P*=1.0 × 10^−7^ for six out of seven) and we proceeded to use these aspects of the 3T3-L1 data as an acceptable proxy for the human insulin-signalling system.

**Figure 3:**
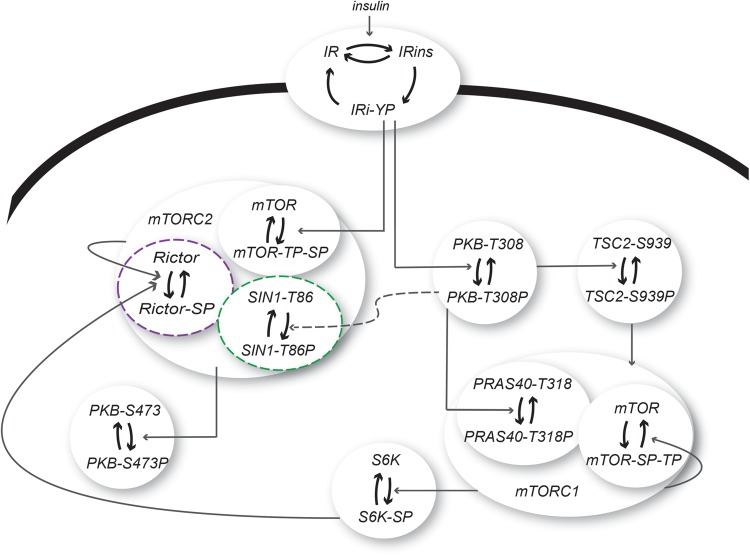
A dynamic mathematical model of mTORC-signalling in 3T3-L1 adipocytes A model for insulin signalling in 3T3-L1 adipocytes with the following proteins: IR that can be phosphorylated at tyrosine residues (Y) and internalized in response to insulin, PKB that can be phosphorylated both on T308 and S473, TSC2, S6K and the protein complexes mTORC1 (with the proteins PRAS40 and mTOR included) and mTORC2 (with the proteins mTOR, rictor and SIN1 included). The tested cross-talk signals to mTORC2 are marked in green (PKB → SIN1) and purple (S6K → rictor). The complete model structure can be found in the Supplementary material.

**Figure 4:**
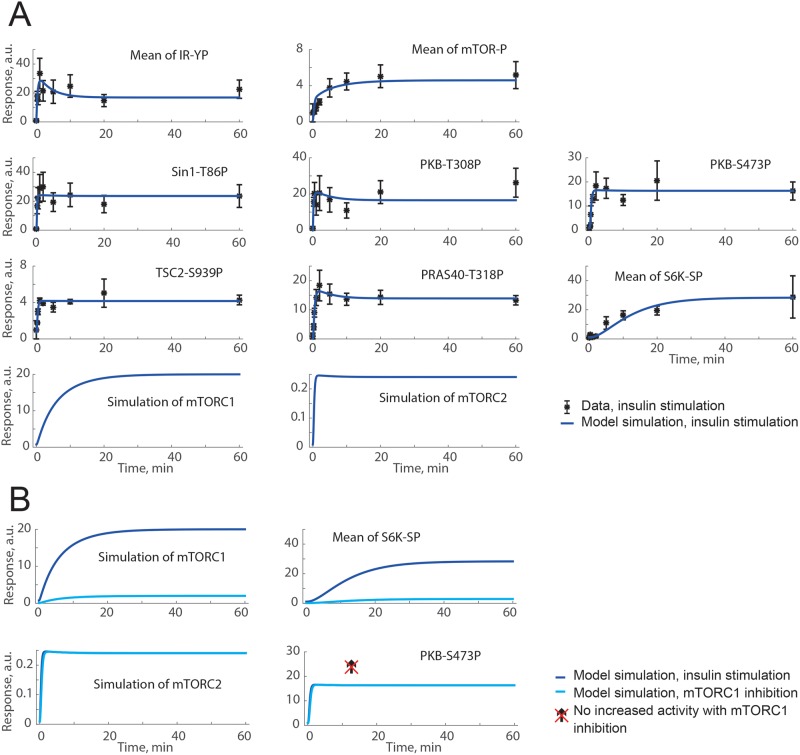
Both a positive and a negative signal from PKB to SIN1-T86P can explain time-course data, but not the increase in PKB-S473P under inhibition of mTORC1 with rapamycin (**A**) Model simulations of insulin stimulation (blue lines) are in agreement with extracted data from [11] (black dots ± S.E.M.). (**B**) Model simulations for insulin stimulation (blue lines) compared with model simulation of mTORC1 inhibition with rapamycin (cyan lines). The crossed over arrow indicates that an increase in PKB-S473P with mTORC1-inhibition is not possible to achieve with this model structure since no signals from mTORC1 to PKB-S473P are included. Shown are the model simulations for a positive signal from PKB to SIN1-T86P. Model simulations with a negative signal were similar (not shown).

Next, the data from 3T3-L1 adipocytes [11] served as a basis in the development of a minimal model of mTORC-signalling in response to insulin (Figure 3). This minimal model consists of three states of IR, corresponding to inactive, autophosphorylated and internalized IR (Figure 3), to capture the dynamic behaviour with a phosphorylation overshoot in the IR-YP data. We have previously shown that such a three-state model of IR can produce this overshoot [6,18,19]. Downstream of IR, we simplified the model by not including all signalling intermediaries, but instead implemented a direct signal from IR-YP to PKB-T308P. This simplification was possible since the time-series data for PKB-T308P and IR-YP had similar dynamic profiles (Figure 4A). In the model, IR-YP also activates mTORC2, which subsequently phosphorylates PKB at Ser^473^. We allowed PKB to be independently phosphorylated at Ser^473^ and Thr^308^ (Figure 3) in order to avert the same unrealistic model behaviour as found in the original human model. Activated PKB then transmits the signal to tuberous sclerosis complex 2 (TSC2) and mTORC1.

We challenged the minimal model of mTORC-signalling to test if the model can simulate the data of the 3T3-L1 cells with PKB phosphorylating SIN1-T86P, as suggested by others [11,12]. To this end, we included this mechanism (Figure 3, green) as a positive or a negative signal from SIN1 to the control of mTORC2 and the subsequent PKB-S473P (Table 1). Both the positive and negative signal produced a model output in agreement with experimental data (Figure 4A), but none could produce the desired increase in PKB-S473P under inhibition of mTORC1 (Figure 4B). This is expected since PKB is upstream of mTORC1 (Figure 3). Therefore, neither a positive nor a negative effect from PKB to SIN1-mTORC2 was sufficient to explain the original observations in diabetic human adipocytes (Table 1).

**Figure 5:**
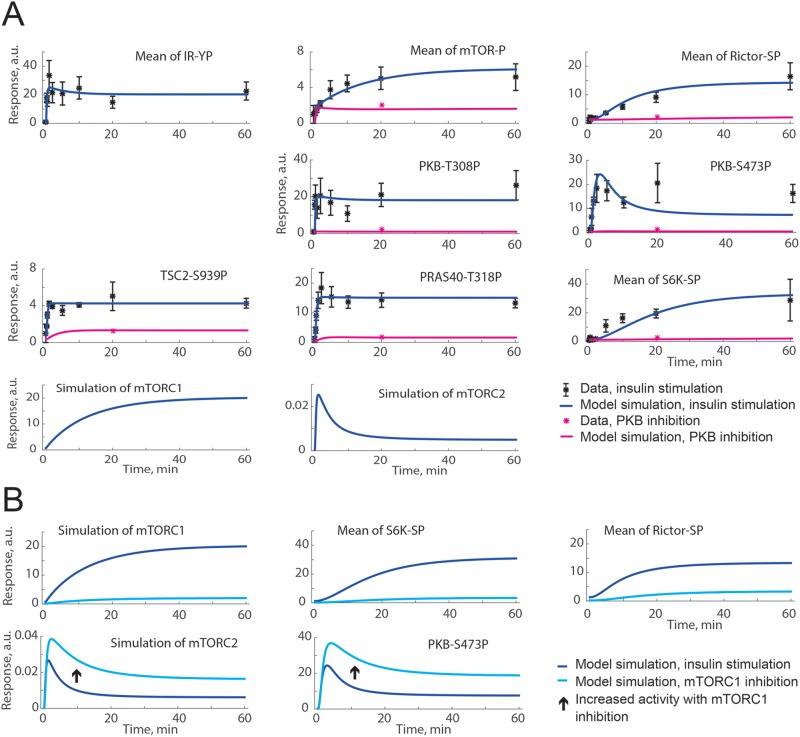
A negative signal from S6K to rictor can explain both time-course data and an increase in PKB-S473P under inhibition of mTORC1 (**A**) Model simulations of insulin stimulation (blue lines) are in agreement with extracted data from [11] (black dots ± S.E.M.). Also shown is the agreement between model simulations of PKB inhibition (implemented as reduced PKB activation; pink lines) and corresponding data from [11] (pink dots) using MK2206 to inhibit PKB. (**B**) Model simulations for insulin stimulation (blue lines) compared with model simulation of mTORC1 inhibition (cyan lines). The arrow shows the simulated increase in mTORC2 activity and PKB-S473P with inhibition of mTORC1.

We then challenged the minimal model of mTORC-signalling by testing a proposed inhibitory signal to mTORC2 from S6K via phosphorylation of rictor [14–16]. The suggested phosphorylation site in rictor, Thr^1135^, was present in the database but only a single repeat had been recorded [11]. We therefore used a combination of three phosphorylation sites in rictor, which had been repeatedly captured and exhibited a clear response to insulin (Ser^1173^, Ser^1176^, and Ser^1478^ ). The negative signal from S6K to rictor–mTORC2 was thus included in the model (Figure 3, purple) with a resulting overall good fit between model simulations and data (Figure 5A, blue). To challenge the model structure, we also tested inhibition of PKB *in silico*, implemented as reduced activation of PKB, again with a qualitative agreement with the corresponding data sets of PKB inhibition in 3T3-L1 adipocytes [11] (Figure 5A, pink). This negative signal S6K → rictor–mTORC2 could also produce an increase in PKB-S473P in response to inhibition of mTORC1 (Figure 5B), and we thus continued to examine this negative signal in the original model of the insulin-signalling network in human adipocytes [1].

### Cross-talk from both PKB–SIN1 and S6K–rictor are required in the human adipocyte model

**Figure 6:**
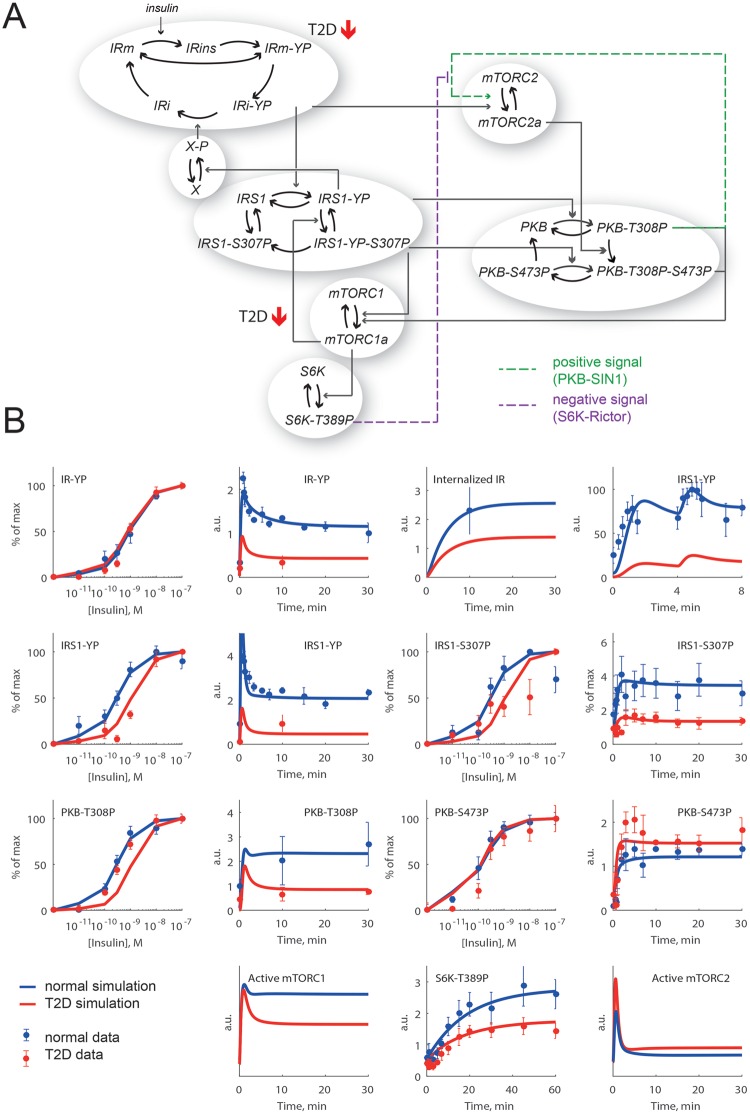
Implementation of combined positive and negative inputs to mTORC2 is required in the human adipocyte model to give a better fit to PKB-S473P data in the diabetic state (**A**) The new diabetes model structure with two branches of signalling downstream IR: IRS1 → S6K that is reduced in diabetes and mTORC2 → PKB-S473P that is increased in diabetes. The model structure includes a positive signal via IRS1-S307P, a negative signal from PKB-S473P to IR and the diabetes parameters from [1] (red arrows). The combined positive (purple dashed arrow) and negative (green dashed arrow) inputs to mTORC2 are the new parts of the model structure. (**B**) Model simulations of the normal (blue lines) and diabetic (red lines) responses to insulin, compared with data (dots ± S.E.M.) from human adipocytes.

We first applied the negative S6K → rictor → mTORC2 signal to the original model (Figure 2A) of insulin signalling in human adipocytes (Figure 6A, purple dashed lines). The signal was implemented to directly inhibit mTORC2. This simplification was done since time-series data are not available for phosphorylation of rictor in human adipocytes. This negative signal was, however, not enough to provide a good fit with all data from human adipocytes, and we next combined this negative signal with the PKB → SIN1 → mTORC2 positive signal (Figure 6A, green dashed lines). We chose a positive signal since it has been shown to be of importance in several cell lines, including 3T3-L1 [20]. For the model with combined negative and positive inputs, there was a good agreement between model simulations and the experimental data of human adipocytes, both normally and in the diabetic state (Figure 6B).

**Figure 7:**
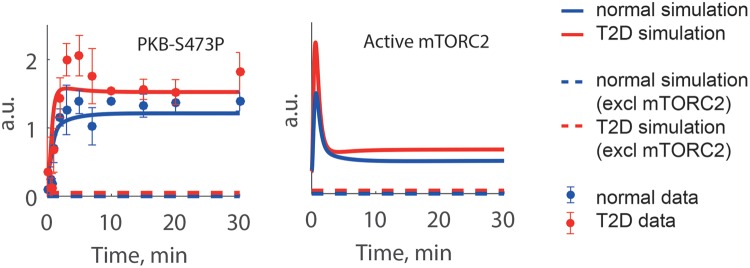
Effect of mTORC2 inhibition in the extended human model The PKB-S473P response is dependent on mTORC2 activity as seen when mTORC2 is inhibited in the model (dashed lines). The PKB-S473P response is totally abolished when mTORC2 is inhibited, both in the diabetic (red) and normal (blue) state.

To confirm the relevance of the new model with cross-talks from S6K and PKB to mTORC2, we predicted the response to inhibition of mTORC2 (Figure 7). The model developed herein responds as expected, i.e. a total inhibition of mTORC2 produces a total inhibition of the PKB-S473P. We could thus extend the model for insulin signalling in human adipocytes to mimic also the increased response of PKB-S473P in the diabetic state.

## Discussion

The present study addresses a missing link in the understanding of altered insulin signalling in cells from diabetic patients, as summarized in a continuously developed mathematical model [1,2,6]. A model that until now, failed to explain an increased insulin response observed for PKB phosphorylated at Ser^473^ in cells from diabetic patients (as compared with healthy controls). PKB is a central protein in the insulin-signalling network, and the increase in response to insulin in the diabetic state might be linked to the disease progression. We have herein studied possible mechanism behind the increased PKB-S473P in human adipocytes, using a new set of more detailed phosphoproteomics data obtained in 3T3-L1 adipocytes. From a selection of these data, we used a rejection-based hypothesis testing approach on cross-talk mechanisms that have been proposed in the literature (Table 1). With this approach, we showed that a combined positive (from PKB [11,12]) and negative (from S6K [12,13]) cross-talk to mTORC2 in the insulin-signalling network can accurately describe the previously unexplained increased PKB-S473P in response to insulin in insulin-resistant human adipocytes from patients with Type 2 diabetes (Figure 6). The results herein are yet another reminder of our present ignorance of feedback and cross-talk signals in the insulin-signalling network as well as in other signalling networks.

The observed increased activity of mTORC2 and subsequent increased PKB-S473P have possible implications for the mechanism of insulin resistance in humans. PKB-S473P phosphorylates the transcription factor FOXO1, which is inactivated upon phosphorylation [21]. Therefore, the increased signalling through this branch enhances inhibition of FOXO1, with consequential effects on transcriptional regulation by this transcription factor. Hence, long-term effects in the diabetic state may be, for example, reduced transcription of IR [22], which is typical of the diabetic state [23,24]. However, it should be noted that the reduced abundance of FOXO1 in the diabetic state may make any enhancement of the insulin effect moot [5].

We used data from a phosphoproteomics database with dynamic phosphorylation data obtained after stimulation of 3T3-L1 adipocytes with insulin [11] to test different mechanisms that could explain an increased PKB-S473P when mTORC1 is inhibited (to mimic the diabetic state of human adipocytes). The 3T3-L1 cell line is differentiated into an adipocyte-like phenotype, but it is different from human adipocytes since it is an immortalized cell line derived from non-human cells (mouse fibroblasts). However, the insulin-signalling pathway is well conserved [25], and the dynamics of the insulin response were largely similar in 3T3-L1 adipocytes and human adipocytes. Examples of this similarity can be seen in the rapid overshoot behaviour of IR phosphorylation and in the slow insulin response for phosphorylation of S6K (3T3-L1 adipocytes in Figure 4A and human adipocytes in Figure 6B). Despite this similarity, it is always paramount that data from different cell types or species are not mixed and used simultaneously to calibrate a mathematical model. With mixing of data, it is not possible to draw conclusions about signalling mechanisms in any of the cell types involved: in such situations, one can no longer use a disagreement between model simulations and data as a basis for rejection of the mechanistic hypotheses implemented by the model – the disagreement can always be due to differences between the cell types. Hence, we used the data from 3T3-L1 cells only to test possible mechanisms, and then verified the found mechanism in a model of insulin signalling in human adipocytes, which only uses data from human adipocytes.

Care should be taken when using experimental model systems to draw conclusions regarding human mechanisms of disease, since the mechanisms might not be the same. One such example is the activity of mTORC1, which has been shown to be hyperactive in high fat-fed rodents [26], but is hypoactive in human adipocytes from diabetic patients [1,3,27,28]. Moreover, phosphorylation of IRS1 at Ser^312^ (human sequence) has been shown to down-regulate insulin signalling in several experimental setups [29–31]. In contrast, knockin mice having IRS1 with alanine replacing Ser^312^ develop severe insulin resistance [32], which strongly refutes phosphorylation of IRS1 at Ser^312^ mediating a negative feedback.

Our developed models are simplified in relation to the underlying biology. One such simplification is that we only consider binding, transport and phosphorylation/dephosphorylation reactions. This means that we use protein phosphorylation as a proxy for protein activity. For many proteins, such as mTORC1 and mTORC2, the true activation pattern is probably more complex [33]. Because of those simplifications, our interaction graphs, e.g. Figure 6A, do not necessarily describe elementary reactions, and our parameters are to be considered as lumped quasi-phenomenological parameters, encapsulating the joint effect of several non-modelled processes. Such simplifications are possible in systems biology modelling that is driven by experimental measurements, where simple and minimal models make it possible to draw conclusions from data. The models herein are minimal, meaning that they only include the mechanisms needed to satisfactorily describe the data. In other words, each submodule in our model could potentially be expanded with a more detailed version, as we have already demonstrated for the IR module [34,35]. We encourage researchers to use our developed models (available in the Supplementary material) and expand with details of their specific research questions regarding insulin resistance.

Our main finding, that a combination of specific positive and negative signals to mTORC2 can explain the difference in insulin signalling at PKB-S473P in diabetic compared with normal non-diabetic human adipocytes, is important in diabetes research and drug development. The combined effect of these negative and positive signals significantly enhances the PKB-S473P by mTORC2 in the diabetic insulin-resistant state when the activity of the negative feedback – from S6K – is much reduced. This finding results from our workflow where proteomics-generated data sets obtained from a cell line are used to identify mechanisms of signalling, where after these mechanisms are verified in a model based on a different data set obtained in primary human cells. This project is a clear example of how an unbiased proteomics approach can support a directed knowledge-based approach. The workflow presented here can be applied in other areas of research in order to get maximum benefit from gathered data in the form of increased mechanistic understandings.

## Materials and methods

### Phosphoproteomic data selection

Phosphoproteomic data from 3T3-L1 adipocytes were derived from the Supplementary Table S2 in [11], which describes the response to 100 nM insulin. We chose data from proteins that have previously been implemented in our studies or that are part of the mTOR complexes. We focused on signalling downstream of the IRS1 and did not include this protein in the analysis of the 3T3-L1 data. In our handling, data were linearized from log 2 space, and mean ± S.E.M. calculated from the available experimental repeats (*n*=3). The data for SIN1 contained only a single repeat, and we therefore estimated S.E.M. to be one-third of the mean of the corresponding measurement. Moreover, phosphorylation sites that correspond to the sites in the previously developed human model of Type 2 diabetes [1] were chosen. Phosphorylation sites not included in the previous model (i.e. mTOR, rictor, SIN1, PRAS40 and TSC2) were chosen based on two criteria: (i) sites that are known in the literature to be involved in insulin signalling and (ii) sites that had a large response to insulin and identified in the inhibition screens (Supplementary Table S1 in [11]). For one protein (S6K), there were no phosphorylation sites in the database that corresponded to the ones used in the previous model [1]. Therefore, the sites used for S6K were chosen according to (ii).

### Human data selection

Data from human adipocytes were extracted from [36]. We selected data corresponding to the stripped-down model structure, i.e*.* IR, IRS1, PKB and S6K (Figure 6B). In the time-series experiments, cells have been treated with 10 or 100 nM insulin and measured over several time points during 30 or 60 min. In the dose-dependent experiments, cells have been treated with different doses of insulin (0.01 to 100 nM) and the response measured after 10 or 30 min. Details on the experiments are found in [36].

### Mathematical modelling

All mathematical analyses, i.e. correlation analysis, simulations and optimizations, were carried out in MATLAB, using the SBtoolbox2 package [37,38]. Since the data from human and 3T3-L1 adipocytes were not collected for the same time points, linear interpolation of the human data was applied in the correlation analysis. In the model analysis, minimal models based on ordinary different equations (ODE) were constructed. Model reactions were assumed to follow mass-action kinetics. An example of ODEs from the models is shown in (eqn 1) and all model equations are found in the Supplementary material together with scripts for model simulation.

(1) $$\begin{array}{c} \frac{d\left(SIN1\right)}{dt}=-\left(k_{1}*SIN1*PKB^{T308P}\right)+\left(k_{2}*SIN1^{T86P}\right)\\ \frac{d\left(SIN1^{T86P}\right)}{dt}=\left(k_{1}*SIN1*PKB^{T308P}\right)-\left(k_{2}*SIN1^{T86P}\right)\\ SIN1\left(0\right)=f\left(insulin=0\right)\\ SIN1^{T86P}\left(0\right)=f\left(insulin=0\right) \end{array}$$

Here, SIN1 is the non-phosphorylated state of the protein, SIN1-T86P is the phosphorylated state, PKB-T308P is the kinase for SIN1, and *k*_1_ and *k*_2_ are the rate constants. Rate constants are referred to as parameters, i.e. values that change the model output. The expression *f(insulin =0)* denotes that the initial conditions were given by the steady-state of the model prior to insulin stimulation, in order to mimic experimental conditions. The same parameter values were used in both the simulation to steady state, as well as in the simulation with insulin.

Estimations of the model parameters were made using the simulated annealing optimization algorithm in SBtoolbox2, minimizing the least square error *V*(*p*) as stated in (eqn 2).

(2) $$V\left(p\right)=\;\underset{i}{\sum }\frac{{\left(y_{i}\left(t\right)-{\hat{y}}_{i}\left(t,p\right)\right)}^{2}}{SEM_{i}^{2}\left(t\right)}$$

Here, *i* denotes measured model variables, i.e. protein phosphorylation, *t* denotes measured time points and *p* denotes model parameters. Additionally, *y* is the measured protein phosphorylation, $\hat{y}$ is the simulated variable value and S.E.M. For the initial analysis with the original human adipocyte model [1], the least square error for PKB-S473P was given an extra weight. The optimization algorithm used, simannealingSBAOClustering, as part of the SBtoolbox2, searches both locally and globally when fitting the model output to data.

## Abbreviations

IR: insulin receptor
IRS1: IR substrate-1
mTOR: mammalian target of rapamycin
mTORC1: mTORC complex 1
mTORC2: mTORC complex 2
PKB: protein kinase B
PKB-S473P: phosphorylation of PKB at Ser^473^
S6K: p70 S6 kinase
TSC2: tuberous sclerosis complex 2
